# Factors influencing return to work after surgery for ulnar nerve compression at the elbow

**DOI:** 10.1038/s41598-022-26363-z

**Published:** 2022-12-23

**Authors:** Alice Giöstad, Malin Zimmerman, Ilka Anker, Erik Dahlin, Lars B. Dahlin, Erika Nyman

**Affiliations:** 1 Department of Biomedical and Clinical Sciences, Linköping University, Linköping, Sweden; 2 Department of Translational Medicine-Hand Surgery, Skåne University Hospital, Lund University, 205 02 Malmö, Sweden; 3 Department of Orthopaedics, Helsingborg Hospital, Helsingborg, Sweden; 4 Department of Hand Surgery, Skåne University Hospital, Malmö, Sweden; 5 Department of Hand Surgery, Plastic Surgery and Burns, Linköping University Hospital, Linköping, Sweden

**Keywords:** Musculoskeletal system, Orthopaedics, Health care economics

## Abstract

Ulnar nerve compression at the elbow (UNE) frequently affects people of working age. Surgically treated patients may not immediately return to work (RTW) postoperatively. In 2008, the Swedish Social Insurance Agency reformed the national insurance policy. We aimed to examine RTW postoperatively for UNE, variations among surgical methods, and potential risk factors for prolonged RTW (sick leave > 6 weeks). Surgically treated cases of UNE (n = 635) from two time periods (2004–2008 and 2009–2014) and two healthcare regions (Southern and South-eastern) were studied retrospectively regarding age, sex, comorbidities, occupation, type of surgery and time to RTW. A sub-analysis of the exact number of weeks before RTW (n = 201) revealed longer RTW for unemployed cases compared to employed cases. Prolonged RTW was seen among younger, manual workers and after transposition or revision surgery. Prolonged RTW was approximately four times more likely after transposition than after simple decompression. Comparisons before and after 2008 showed occupational differences and differences in RTW, where cases operated before 2008 more often had permanent sickness benefit, but the reform of the social insurance system did not seem to influence RTW. In conclusion, unemployment, younger age at surgery, manual labour, transposition, and revision surgery were related to prolonged RTW.

## Introduction

Ulnar nerve compression at the elbow (UNE) is the second most common peripheral entrapment neuropathy in the upper extremity and most often affects people of working age^1^. Two entities of this condition, entrapment beneath the humeroulnar aponeurotic arcade and external compression of the ulnar nerve in the retroepicondylar groove, have been identified, comprising patient groups with varied preoperative characteristics and type of work^2^. Surgically treated patients may not immediately return to work (RTW) after surgery, depending on the patient’s work tasks, since the operated arm needs to heal and functionally recover. In Sweden, the right to medical insurance compensation during sick leave, called sickness benefit, is dependent on a sickness certificate issued by the treating physician. This is required if the sickness period exceeds seven days. For those in employment, the employer pays the first 14 days of sick pay, after which the Swedish Social Insurance Agency pays sickness benefit. Unemployed people also receive sickness benefit. Permanent sickness benefit can be paid if a person, aged 19–64 years, affected by disease, trauma, or disability, is judged to be currently or permanently unable to work^3^.

The Swedish Social Insurance Agency reformed the social insurance system in 2008, because of parliamentary decisions, introducing a so-called rehabilitation chain^4,5^. The reform resulted in more stringent rules for the right to sickness benefit, and rejections became more common in cases of longer periods of sick leave. Additional information regarding the social insurance system and the rehabilitation chain is presented in Table 1. How this reform of the social insurance system affected the pattern of sick leave and RTW in various hand disorders has not previously been highlighted.

**Table 1:** Information about the Swedish social insurance system.

An assessment of the affected person’s inability to work, based on the current disease or condition in relation to their work activities, is given in the sickness certificate. Sickness certificates are sent to the Swedish Social Insurance Agency, which approves or rejects insurance according to current scientifically based insurance medicine guidelines in relation to the sickness certificate Briefly, the changes in the rehabilitation-chain included set time-limits for reviewing a person’s eligibility to sickness benefit and for judging their ability to RTW both in their original work and in all available occupations*.* Sick leave guidelines were also revised to promote RTW, and a maximum total duration for government financed sick leave was introduced	

*RTW* return to work.

The sick leave pattern and time to RTW after surgery for UNE has not been studied as thoroughly as RTW after carpal tunnel release (CTR), traumatic ulnar nerve injuries or severe hand trauma^6–8^. The definition of return to work may vary among studies, comprising both return to part time work or return to work with modified work duties. Several predictive factors for prolonged RTW after CTR have been identified, including manual labour, depression, and bilateral surgery^6,9,10^. Other factors such as being self-employed, not being able to return to modified duties and not receiving workers’ compensation on the contrary have the opposite effect and may promote earlier RTW^6^. Worse preoperative status among patients with carpal tunnel syndrome (CTS), evaluated by the short version of Disabilities of the Arm, Shoulder and Hand, QuickDASH, predicts lower rate of RTW^10^. In addition, a history of long-term sick leave can affect the postoperative outcome measured by the QuickDASH for patients having surgery for UNE^11^.

Our aims in the present study were to examine RTW after surgery for UNE, to map out potential variations among different surgical methods and basic characteristics, and to explore whether RTW was affected by the reform of the social insurance system.

## Methods

Data from cases surgically treated for UNE were retrospectively collected in two different health care regions, during two time periods (Southern region during 2004–2008 and 2010–2011, South-Eastern region 2011–2014). The cases were identified using ICD-10 codes for intervention (KVÅ ACC43 and ACC53). The surgical methods used were simple decompression, anterior subcutaneous transposition, or anterior submuscular transposition. Type of surgery was categorised as simple decompression, ulnar nerve transposition (subcutaneous or submuscular), and revision surgery (secondary transposition or secondary decompression). Conservative treatment of minimum 3 months and patient education is clinical practice at the current clinics and if symptoms remain at that time surgery is considered.

Information concerning age, sex, type of work, comorbidity, type of surgery and weeks before RTW was collected from medical charts and sickness certificates. The type of work was categorized into manual versus non-manual labour, based on the Swedish Standard Classification of Occupations from 2012 (SSYK 2012), which in turn is based on the International Standard Classification of Occupations (ISCO-08)^12^. The same categorization has been used in previous studies^13^. Unemployment, permanent sickness benefit, or retirement were also noted. Cases of sick leave based on other chronic diseases were excluded, since such data could not be judged and interpreted; thus, only sick leave due to ulnar nerve surgery was registered. The time between surgery and return to fulltime work was divided into three periods: < 3 weeks, 3–6 weeks and > 6 weeks. The National Board of Health and Welfare (Socialstyrelsen.se) publishes guidelines for length of sick leave in connection with different diseases/diagnoses; e.g. after CTR the recommended length of sick leave varies between 4 and 8 weeks, depending on the work load^14^. For UNE, directions for sick leave are available solely for conservative treatment^15^. The cut-off for prolonged RTW is based on local clinical experience and according to prevailing practice that most patients can return to work after 6 weeks, regardless of how heavy the work is.

Each treated nerve was considered as a separate case and a discrete statistical entity. The Shapiro–Wilk test of normality was used to control for normal distribution of data. Parametric data are presented as mean (SD); non-parametric as median [IQR]. Nominal data are presented as percentages (n/n). T-tests or ANOVA were used for continuous normally distributed data. The Chi-Square or Mann–Whitney U test were used to compare groups and analyse dichotomized variables, respectively. The Kruskal–Wallis test and Mann–Whitney U tests, respectively, were used subsequently for non-parametric data. A binary logistic regression was conducted to assess the effect of individual factors on prolonged RTW (sick leave > 6 weeks) for those able to work (those receiving permanent sickness benefit, unknown occupation and retirees were excluded). Age, sex, type of occupation, smoking, diabetes, cardiovascular diseases, musculoskeletal diseases, psychiatric diagnoses, other comorbidities (diagnoses/disorders that did not fit the other categories, e.g., pulmonary diseases, cancer, endocrine diagnoses), type of surgery and surgery before or after 2008 were included in the regression and run with the “Forward LR” method.

The Regional Ethics Review Boards in Linköping and Lund, Sweden were applied to for ethical approval. Approval was obtained from the Board in Linköping, (register number 2016/ 88-31); the Committee in Lund (#2011/607) found the study to be sound, without ethical problems, and judged that it was not subject to the relevant law governing research ethics in Sweden. Thus, neither advertising nor formal informed consent from each patient was required. Chief of service at the local department approved the quality control. For these reasons only one formal permission number is attached to the study. The issue was discussed with experts in ethics, who endorsed the conduct of the study following the decision by the Lund Ethics Committee. All methods were carried out in accordance with the pertinent guidelines and regulations. The local and regional ethic committees adhere to the Declaration of Helsinki. Because of the retrospective design, the Regional Ethics Review Board in Linköping and the Ethical Committee in Lund waived the requirement for written informed consent of study participants, data were kept anonymous and confidential.

## Results

### Basic characteristics

In total, 635 cases (348 females and 287 males; mean age at surgery 49 ± 13 years) were included from the two regions, comprising 439 simple decompressions, 109 transpositions (71 subcutaneous and 38 submuscular) and 87 revision surgeries [75 secondary transpositions (38 subcutaneous and 37 submuscular) and 12 secondary decompressions]. When comparing subcutaneous and submuscular transpositions there were no differences regarding age, sex, smoking, or type of occupation. The cases with subcutaneous and submuscular transpositions are therefore grouped together in the following analyses. Musculoskeletal comorbidities were more common among transposition cases (both subcutaneous and submuscular) compared to cases treated with simple decompression (data not shown, Chi^2^, p = 0.036).

Musculoskeletal diagnoses, including shoulder impingement, cervical rhizopathy, other nerve compression disorders and low back pain, were the most common comorbidities (73%, 466/635), followed by cardiovascular diseases (24%, 150/632). Psychiatric diagnoses were noted in 16% (93/574) and daily cigarette smoking in 44% (191/435) of the study population. Details are presented in Table 2. Cases with simple decompression were older than cases with revision surgery (Table 3) For additional differences regarding surgical methods see Table 3.

**Table 2:** Basic characteristics for the whole population and divided based on time before return to work.

		Total population	RTW < 3w	RTW 3–6w	RTW > 6w	Already on sick leave/permanent sickness benefit	Retired
		n/635 (%)	n/75 (%)	n/150 (%)	n/215 (%)	n/97 (%)	n/34 (%)
Sex	Female	348 (55)	29 (39)	82 (55)	121 (56)	64 (66)	31 (48)
	Male	287 (45)	46 (61)	68 (45)	91 (44)	33 (34)	33 (52)
Age at surgery, years (mean ± SD)		48.8 ± 12.9	45.6 ± 13.4	47.1 ± 10.2	44.9 ± 9.9	47.9 ± 10.3	71.3 ± 6.0
Occupation	Non-manual labour	140 (22)	32 (43)	42 (28)	45 (21)	16 (17)	N/A
	Manual labour	279 (44)	19 (25)	84 (56)	124 (58)	40 (41)	N/A
	Unknown	43 (7)	7 (9)	12 (8)	12 (6)	1 (1)	N/A
	Student	13 (2)	8 (11)	1 (1)	2 (1)	0 (0)	N/A
	Unemployed	66 (10)	8 (11)	11 (7)	30 /14)	13 (13)	N/A
	Permanent sickness benefit	30 (5)	1 (1)	0 (0)	2 (1)	27 (28)	N/A
	Retired	64 (10)	N/A	N/A	N/A	N/A	64 (100)
Smoking^a^	Yes	191 (44)	18 (41)	40 (33)	75 (48)	34 (69)	15 (36)
	No	244 (56)	26 (59)	80 (67)	80 (52)	15 (31)	27 (64)
Comorbidity	None	84 (13)	18 (24)	22 (15)	29 (14)	8 (8)	3 (5)
	Diabetes^b^	69 (11)	6 (8)	15 (10)	16 (8)	13 (13)	12 (19)
	CVD^b^	150 (24)	15 (20)	33 (22)	39 (18)	15 (16)	37 (58)
	Musculoskeletal	466 (73)	48 (64)	108 (72)	162 (75)	82 (85)	43 (67)
	Psychiatric^c^	93 (16)	14 (21)	16 (12)	30 (15)	26 (27)	1 (2)
	Other comorbidity	192 (30)	15 (20)	45 (30)	62 (29)	22 (23)	33 (52)
Type of surgery	Simple decompression	439 (69)	67 (89)	124 (83)	109 (51)	66 (68)	50 (78)
	Transposition^d^	109 (17)	6 (8)	18 (12)	53 (25)	13 (13)	9 (14)
	Revision surgery	87 (14)	2 (3)	8 (5)	53 (35)	18 (19)	5 (8)

*W* weeks, *RTW* return to work, *CVD* cardiovascular diseases.

^a^n = 435.

^b^n = 632.

^c^n = 574.

^d^Transpositions include subcutaneous and submuscular ulnar nerve transpositions.

**Table 3:** Comparison of basic characteristics and time before return to work between the three different surgery groups.

		Simple decompression	Transposition^e^	Revision surgery	P-value
		n/439 (%)	n/109 (%)	n/87 (%)	
Sex	Female	229 (52)	60 (55)	59 (68)	**0.028**
	Male	210 (48)	49 (45)	28 (32)	
Age at surgery, years (mean ± SD)		49.9 (12.7)	47.5 (13.1)	45.1 (12.6)	0.004*
Occupation	Non-manual labour	96 (22)	27 (25)	17 (20)	0.506
	Manual labour	189 (43)	47 (43)	43 (49)	0.666
	Unknown	31 (7)	7 (6)	5 (6)	0.957
	Student	9 (2)	1 (1)	3 (3)	0.462
	Unemployed	38 (9)	15 (14)	13 (15)	0.096
	Permanent sickness benefit	26 (6)	3 (3)	1 (1)	0.137
	Retired	50 (11)	9 (8)	5 (6)	0.682
Smoking^a^	Yes	132 (46)	32 (36)	27 (46)	0.279
	No	156 (54)	56 (64)	32 (54)	
Comorbidity	None	66 (15)	12 (11)	6 (7)	0.093
	Diabetes^b^	48 (11)	11 (10)	10 (12)	0.947
	CVD^b^	106 (24)	29 (27)	15 (17)	0.272
	Musculoskeletal	310 (71)	85 (78)	71 (82)	0.052
	Psychiatric^c^	57 (15)	13 (13)	23 (26)	**0.018**
	Other comorbidity	119 (27)	46 (42)	27 (31)	**0.009**
Time to return to full time work^d^	< 3 weeks	67 (16)	6 (6)	2 (2)	** < 0.001**
	3–6 weeks	124 (30)	18 (18)	8 (9)	** < 0.001**
	> 6 weeks	109 (26)	53 (54)	53 (62)	** < 0.001**
	Already on sick leave/permanent sickness benefit	66 (16)	13 (13)	18 (21)	0.342
	Retired	50 (12)	9 (9)	5 (6)	0.203

*CVD* cardiovascular diseases.

P-values represent results from Chi^2^ tests for every variable except age where ANOVA was used. Significant differences are marked in bold.

*Tukey’s HSD test showed a statistically significant difference between simple decompression and revision surgery, p = 0.005.

^a^n = 435.

^b^n = 632.

^c^n = 574.

^d^n = 601.

^e^Transpositions include subcutaneous and submuscular ulnar nerve transpositions.

### Return to work

The time before full RTW after surgery was < 3 weeks in 13% (75/601), 3–6 weeks in 25% (150/601), and > 6 weeks in 36% (215/601) of the study population (Table 2). A total of 27% (161/601) had permanent sickness benefit, were retired or already on sick leave due to other unrelated health issues (Table 2). The time before RTW was longer for those treated with revision surgery or transposition than for those with simple decompression (Table 3). We were unable to retrieve information about sick leave for 5% (34/635) of the cases.

### Cases able to work

**Figure 1:**
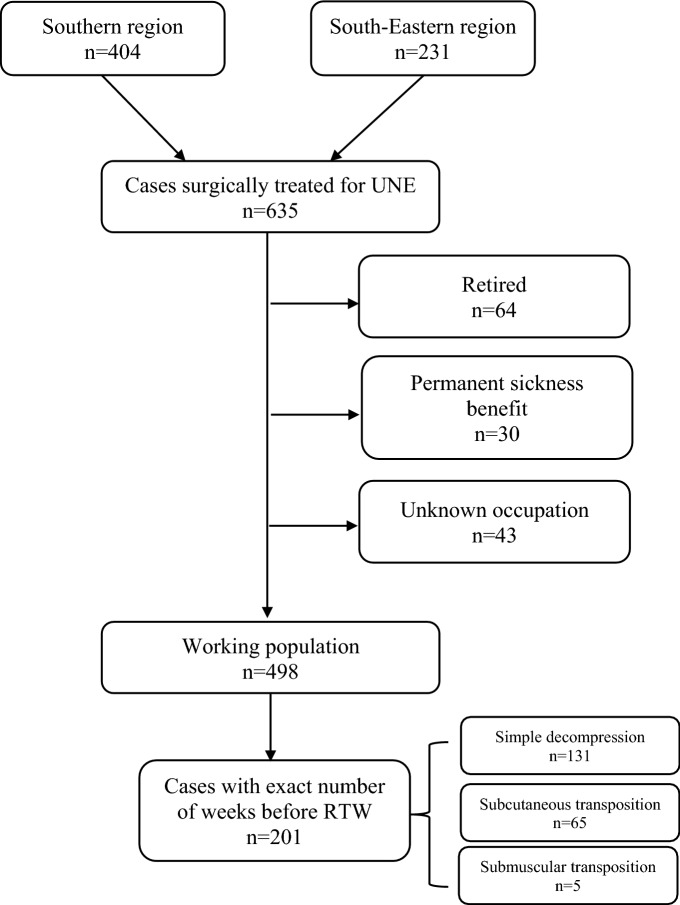
Flowchart over included cases. *UNE* ulnar nerve compression at the elbow, *RTW* return to work.

After excluding cases with permanent sickness benefit, unknown occupation and those who were retired, a total of 498 cases constituted the working population (Fig. 1). Analysing only cases able to work (Fig. 1), 41% (201/498) did not RTW within 6 weeks after surgery. There were no differences regarding sex, smoking status, or comorbidity regarding RTW within 6 weeks after surgery. Cases with prolonged RTW in the working population were younger (age at surgery 44.7 ± 9 vs 46.4 ± 11, p = 0.036), more seldom had simple decompression (Chi^2^, p < 0.001) and more seldom had non-manual labour (Chi^2^, p = 0.013).

### Sub-analysis with exact weeks before RTW

**Figure 2:**
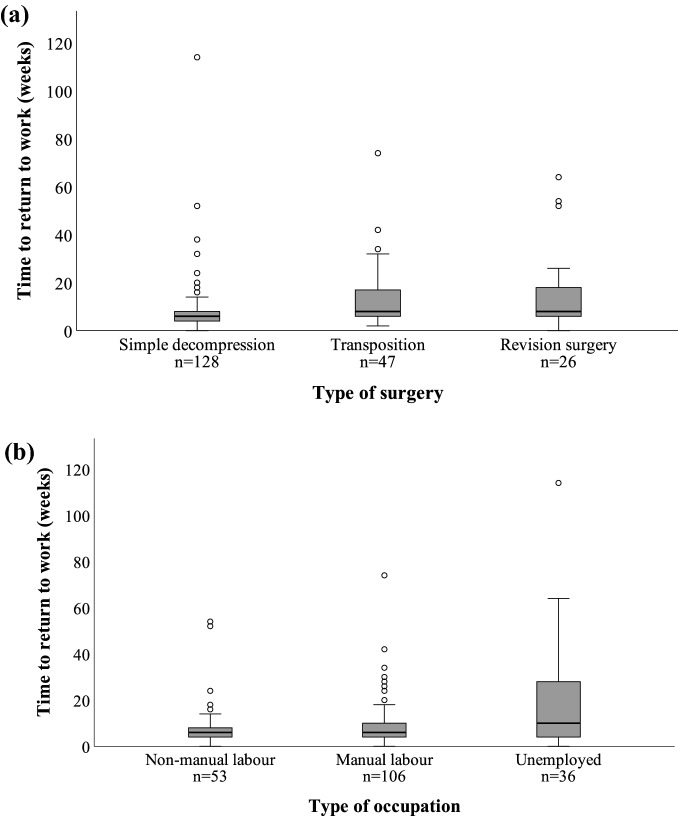
Time to full return to work, weeks. Groups based on (**a**) type of surgery, n = 201 and (**b**) type of occupation (data for unknown employment and students not shown), non-manual labour n = 53, manual labour n = 106, unemployed n = 36. Circles represent outliers. Subcutaneous transpositions and submuscular transpositions are grouped together in (**a**) since there were no differences between the groups.

Information about the exact number of weeks before RTW was obtained in 201 cases from the working population (27 cases from the Southern region and 174 cases from the South-Eastern region; Fig. 1). Median time to RTW was 6 weeks [IQR 4–10]. Cases with simple decompression had the shortest time before RTW; median 6 weeks [IQR 4–8]. The median time before RTW after transposition or revision surgery was 8 weeks [IQR 6–18 and 6–19 respectively] (Table 3; Fig. 2a).

Significant differences were found when types of occupation were compared in this sub-analysis. Unemployed cases had a median of 10 weeks [IQR 4–29] of sick leave, which was longer than for the rest of the working population (Mann–Whitney U test, p = 0.023), where both non-manual and manual labour had a median time before RTW of 6 weeks [IQR 4–8 and 4–10, respectively] (Fig. 2b) (subsequent Mann–Whitney U tests p = 0.214 and p = 0.706, respectively; Fig. 2b).

### Regression analyses for return to work

Conducting a binary logistic regression, controlling for basic characteristics and comorbidities (with simple decompression and non-manual work as reference categories), we found that there was an increased risk of prolonged RTW in cases with either subcutaneous transposition (OR 3.84, 95% CI 2.20–6.69, p < 0.001) or submuscular transposition of the ulnar nerve (OR 3.94, 95% CI 1.83–8.49, p < 0.001). No other statistically significant associations were found for the remaining variables.

### Differences before and after the reform of the social insurance system

When comparing cases operated on before or after 2008, we found several differences (Supplementary Table 1). Concerning occupational differences; before 2008 more cases were already on sick leave, due to another unrelated health issue, or received permanent sickness benefit, and after 2008 a higher proportion were unemployed. A higher proportion were smokers before 2008 than after 2008. Psychiatric diagnoses, cardiovascular diseases, and other comorbidities were all more common after 2008 (Supplementary Table 1).

## Discussion

This study examines RTW after surgery for UNE, comparing different surgical methods during two different time periods, before and after a change in the Swedish national guidelines concerning the sickness benefit system. In the regression model, we found that cases treated surgically with transposition of the ulnar nerve were approximately four times more likely to have prolonged RTW compared to cases treated with simple decompression. The change in the sickness benefit system did not influence prolonged RTW after adjustment for other variables. Before 2008 more cases were already on sick leave due to other unrelated health issues at the time of the ulnar nerve surgery or received permanent sickness benefit. Interestingly, unemployment, manual labour, and younger age at surgery were found to be related to prolonged RTW.

In the sub-analysis, where an exact number of weeks before RTW was available, the unemployed had significantly longer sick leave compared to the rest of the working population, but no association could be shown with prolonged RTW in the regression model when the cut-off for prolonged RTW was set at 6 weeks. However, in a previous study on UNE, being out of work, having a concomitant CTS and being older, predicted a lower chance of RTW in both conservatively and surgically treated groups^16^. For those in the surgically treated group, being out of work at the time of the diagnosis predicted failure to RTW (when adjusted for other clinical, electrophysiological, and sociodemographic factors^16^), which is in accordance with our findings.

In a synthesis of systematic reviews of factors affecting RTW for several different injuries/illnesses, common factors that negatively influence RTW were older age, being female, previous sick leave and being unemployed^17^. In the present study, being unemployed was more common after 2008, but the distribution over the surgery groups was equal, while before 2008 a higher percentage overall were employed. In Sweden, there are different criteria for employed versus unemployed people when assessing whether the payment of sickness benefit is justified. If employed, eligibility for sickness benefit is based on one’s current job and inability to perform regular work tasks, while for the unemployed the right to sickness benefit is judged according to their ability to seek work and perform any job tasks available on the labour market. It is possible, therefore, that the Swedish Social Insurance Agency could deny sickness benefit to unemployed people to a greater extent than to employed people; however, our study shows the opposite, with the unemployed having a longer period of sick leave after surgery. One hypothesis for explaining this discrepancy could be a lack of motivation for the unemployed to terminate their sick leave, compared to the economic incitement for employed patients to return to work. The unemployed could also have a secondary economic gain receiving the sickness benefit. A Swedish employer is obliged to customize the work tasks for the employed based on their disabilities, which can promote earlier RTW, while this is not available for the unemployed. Our findings may also be influenced by which types of work the unemployed cases in our study have capability to take. Greater mental health comorbidity could be another reason^18^. National differences in insurance systems could possibly explain why our results diverge from other studies performed in other contexts^19^. Differences seen before and after 2008 could be influenced by the reform of the social insurance system, but the lack of other possible confounders, such as socioeconomic status, more precise comorbidity, and missing values, precludes the drawing of any such conclusions from our study.

Studies on RTW specifically after surgical treatment for UNE, i.e., non-traumatic ulnar nerve related disorders, are scarce, but RTW has been investigated in other peripheral nerve disorders. Studies on traumatic nerve injuries, as isolated ulnar or median nerve injuries or combined nerve injuries, indicate that higher education level, type of occupation (non-manual labour) and compliance with hand therapy are significant predictors for RTW within a year^7^. This is consistent with our current findings, i.e., patients with manual labour have a prolonged time to RTW. These findings seem logical since return to manual labour might be expected to take longer after surgery in the upper extremity. Patients with manual labour more often have UNE at the humeroulnar aponeurotic arcade, for which early surgical release often is recommended, compared to patients with non-manual work in which UNE at the retroepicondylar groove is more common and initial conservative treatment is of greater value^2^. In our study, however, most of the patients underwent conservative treatment before surgery as part of the clinical routine. In CTS, non-manual labour, self-employment, not receiving workers’ compensation and the ability to modify work duties were in a systematic review associated with earlier RTW^6^. Poorer pre-operative hand function and manual labour reduce the likelihood of RTW within a year after surgery for CTS^10^.

Smoking at the time of the surgery was more common before 2008. In a previous study, we found that smoking is a risk factor for complications after surgery^20^. In a Finnish birth cohort, there was an increased risk of UNE among patients with physically demanding work and patients working with vibrating tools and in changing temperatures, and smoking increased the effects of work-related risk factors^21^. However, in the regression model, smoking did not influence RTW.

Psychiatric comorbidity was relatively common in our study, affecting 16% of the population. In a large Swedish population-based registry study, individuals with surgically treated UNE had an increased risk of impaired psychological health measured by the use of psychotropic drugs (i.e., antidepressants, psycholeptics or psycholeptics and psychoanaleptics in combination) than individuals without UNE^22^.

The only significant contributing factor for prolonged RTW in our regression model was type of surgery, where both subcutaneous and submuscular transpositions were associated with prolonged RTW. A cost-analysis showed that the time before RTW was longer for patients who underwent anterior subcutaneous transposition than for patients having simple decompression and longer for patients in paid work compared to those in unpaid work (i.e., housewives)^23^. Long-term sick leave before surgery negatively affected outcome after surgery for UNE in a large registry-based study^11^ . Compared to the general Swedish population, patients with UNE were socioeconomically deprived^11^. For CTS, prolonged sick leave after surgery (> 39 days) was associated with a higher postoperative score on the short version of Disability of the Arm, Shoulder and Hand (QuickDASH), indicating a subjective poorer surgical outcome^13^. Hence, socioeconomic factors seem to be important for both the prevalence of ulnar nerve compression and the results following treatment for it.

One weakness of this study is that we were not able to analyse the extent of part-time sick leave, which may have altered the results. In Sweden, sick leave can be fulltime or part-time (75%, 50% or 25%) based on the doctor’s assessment of the patient´s ability to work in relation to their disability. In addition, we only had data on the exact length of sick leave for 35% of the study population. Another problem with the Swedish insurance system is that patients themselves can discontinue the time-specified sickness certificate issued by the treating doctor and RTW without reporting back to either the treating doctor or clinic. Another limitation is the lack of pre- and postoperative status/grading of the severity of the ulnar nerve compression and patient reported outcome measures, which also may influence the time before RTW, as well as more precise socioeconomic data. Prospective studies with more detailed pre-operative information about symptoms, ulnar nerve compression severity, part-time sick leave, and exact time before RTW after surgery are needed. On the other hand, the strength of this study is the comparatively large number of cases included. The absence of exclusion based on comorbidity makes this study applicable in clinical reality. Having cases from two different healthcare regions also provides a broader perspective on the heterogeneous population with UNE.

In this study, we examined the time before RTW after surgery for UNE from several different aspects, by combining data from two different healthcare regions in Sweden, enabling analysis of the effect of the social reform of the Swedish Social Insurance Agency in 2008, as well as analyses on the surgical methods, and related patient characteristics. We conclude that individuals treated with transposition of the ulnar nerve are at a greater risk of having prolonged RTW than individuals treated with simple decompression. Other risk factors for prolonged RTW include younger age, unemployment and manual labour. Further, the change in national insurance policy in 2008 did not seem to influence time before RTW, when adjusted for other confounders, but the demographics differ in several ways before and after 2008.

## Supplementary Information

Supplementary Table 1.

## Data Availability

The data that support the findings of this study are available from Region Östergötland and Region Skåne, but restrictions apply to the availability of these data, which were used under license for the current study, and so are not publicly available. Data are, however, available from the authors upon reasonable request and with permission of the Regional Ethics Review Board in Linköping, the Ethical Committee in Lund and Region Skåne.
