# Supplementary material for: Factors influencing return to work after surgery for ulnar nerve compression at the elbow
Source: Sci Rep. 2022 Dec 23;12:22229. doi: 10.1038/s41598-022-26363-z (PMC9789024; doi:10.1038/s41598-022-26363-z)
Supplement: Supplementary file 1 — Supplementary Table 1. [file 41598_2022_26363_MOESM1_ESM.docx]

**Supplementary table 1.** *Basic characteristics and time before return to work for the whole study population and comparison between cases operated on before and after 2008.*

|  | | **Total population** | **Surgery before 2008** | **Surgery after 2008** |  |
| --- | --- | --- | --- | --- | --- |
|  |  | **n/635 (%)** | **n/343 (%)** | **n/292 (%)** | **P-value** |
| Sex | Female | 348 (55) | 176 (51) | 172 (59) | 0.055 |
|  | Male | 287 (45) | 167 (49) | 120 (41) |  |
| Age at surgery, years (mean ± SD) | | 48.8 ±12.9 | 48.5 ± 12.2 | 49.1 ± 13.7 | 0.575 |
| Occupation | Non-manual labour | 140 (22) | 83 (24) | 57 (20) | **0.026** |
|  | Manual labour | 279 (44) | 161 (47) | 118 (40) | **0.047** |
|  | Unknown | 43 (7) | 15 (4) | 28 (10) | **0.021** |
|  | Student | 13 (2) | 6 (2) | 7 (2) | 0.566 |
|  | Unemployed | 66 (10) | 21 (6) | 45 (15) | **<0.001** |
|  | Permanent sickness benefit | 30 (5) | 23 (7) | 7 (2) | **0.007** |
|  | Retired | 64 (10) | 34 (10) | 30 (10) | **0.006** |
| Smoking^a^ | Yes | 191 (44) | 91 (63) | 100 (35) | **<0.001** |
|  | No | 244 (56) | 54 (37) | 190 (66) |  |
| Comorbidity | None | 84 (13) | 54 (16) | 30 (10) | **0.043** |
|  | Diabetes^b^ | 69 (11) | 32 (9) | 37 (13) | 0.163 |
|  | CVD^b^ | 150 (24) | 67 (20) | 83 (29) | **0.007** |
|  | Musculoskeletal | 466 (73) | 242 (71) | 224 (77) | 0.080 |
|  | Psychiatric^c^ | 93 (16) | 41 (12) | 52 (23) | **0.001** |
|  | Other comorbidity | 192 (30) | 51 (15) | 141 (48) | **<0.001** |
| Type of surgery | Simple decompression | 439 (69) | 248 (72) | 191 (65) | 0.061 |
|  | Transposition^1^ | 109 (17) | 43 (13) | 66 (23) | **0.001** |
|  | Revision surgery | 87 (14) | 52 (15) | 35 (12) | 0.246 |
| Time before return to work^d^ | <3 weeks | 75 (13) | 45 (14) | 30 (11) | 0.410 |
|  | 3-6 weeks | 150 (25) | 55 (17) | 95 (36) | **<0.001** |
|  | > 6 weeks | 215 (36) | 118 (35) | 97 (36) | 0.799 |
|  | Already on sick leave/permanent sickness benefit | 97 (16) | 82 (25) | 15 (6) | **<0.001** |
|  | Retired | 64 (11) | 34 (10) | 30 (11) | 0.677 |

CVD = cardiovascular diseases

^1^Transpositions include subcutaneous and submuscular ulnar nerve transpositions.

P-values represent results from Chi2 tests and t-test (age variable) comparing surgery before 2008 and surgery after 2008. Significant differences are marked in bold.

^a^n= 435, ^b^n=632, ^c^n=574, ^d^n= 601
